# Venous thromboembolism after total joint arthroplasty: results from a Japanese multicenter cohort study

**DOI:** 10.1186/ar4616

**Published:** 2014-07-21

**Authors:** Kiyoshi Migita, Seiji Bito, Mashio Nakamura, Shigeki Miyata, Masanobu Saito, Hirosi Kakizaki, Yuichiro Nakayama, Tomohiro Matsusita, Itaru Furuichi, Yoshihiro Sasazaki, Takaaki Tanaka, Mamoru Yoshida, Hironori Kaneko, Isao Abe, Takatomo Mine, Kazuhiko Ihara, Shigeyuki Kuratsu, Koichiro Saisho, Hisaaki Miyahara, Tateki Segata, Yasuaki Nakagawa, Masataka Kamei, Takafumi Torigoshi, Satoru Motokawa

**Affiliations:** 1 Japanese National Hospital Organization (NHO)-EBM study group; Japanese study of Prevention and Actual situation of Venous Thromboembolism after Total Arthroplasty (J-PSVT), Higashigaoka 2-5-21, Meguro, Tokyo 152-8621, Japan; 2 Division of Clinical Epidemiology, NHO Tokyo Medical Center, Higashigaoka 2-5-1, Meguro, Tokyo 152-8902, Japan; 3 Department of Clinical Cardiovascular Research, Mie University Graduate School of Medicine, Edohashi 2-174, Tsu, Mie 514-8507, Japan; 4 Division of Transfusion Medicine, National Cerebral and Cardiovascular Center, Fujishirodai 5-7-1, Suita, Osaka 565-8565, Japan; 5 Department of Anesthesiology, National Cerebral and Cardiovascular Center, Fujishirodai 5-7-1, Suita, Osaka 565-8565, Japan; 6 Department of Orthopedic Surgery, NHO Nagasaki Medical Center, Kubara 2-1001-1, Omura, 856-8652, Japan

## Abstract

**Introduction:**

Real-world evidence of the effectiveness of pharmacological thromboprophylaxis for venous thromboembolism (VTE) is limited. Our objective was to assess the effectiveness and safety of thromboprophylactic regimens in Japanese patients undergoing joint replacement in a real-world setting.

**Method:**

Overall, 1,294 patients (1,073 females and 221 males) who underwent total knee arthroplasty (TKA) and 868 patients (740 females and 128 males) who underwent total hip arthroplasty (THA) in 34 Japanese national hospital organization (NHO) hospitals were enrolled. The primary efficacy outcome was the incidence of deep vein thrombosis (DVT) detected by mandatory bilateral ultrasonography up to post-operative day (POD) 10 and pulmonary embolism (PE) up to POD28. The main safety outcomes were bleeding (major or minor) and death from any cause up to POD28.

**Results:**

Patients undergoing TKA (n = 1,294) received fondaparinux (n = 360), enoxaparin (n = 223), unfractionated heparin (n = 72), anti-platelet agents (n = 45), or no medication (n = 594). Patients undergoing THA (n = 868) received fondaparinux (n = 261), enoxaparin (n = 148), unfractionated heparin (n = 32), anti-platelet agents (n = 44), or no medication (n = 383). The incidence rates of sonographically diagnosed DVTs up to POD10 were 24.3% in patients undergoing TKA and 12.6% in patients undergoing THA, and the incidence rates of major bleeding up to POD28 were 1.2% and 2.3%, respectively. Neither fatal bleeding nor fatal pulmonary embolism occurred. Significant risk factors for postoperative VTE identified by multivariate analysis included gender (female) in both TKA and THA groups and use of a foot pump in the TKA group. Only prophylaxis with fondaparinux reduced the occurrence of VTE significantly in both groups. Propensity score matching analysis (fondaparinux versus enoxaparin) showed that the incidence of DVT was lower (relative risk 0.70, 95% confidence interval (CI) 0.58 to 0.85, *P* = 0.002 in TKA and relative risk 0.73, 95% CI 0.53 to 0.99, *P* = 0.134 in THA) but that the incidence of major bleeding was higher in the fondaparinux than in the enoxaparin group (3.4% versus 0.5%, *P* = 0.062 in TKA and 4.9% versus 0%, *P* = 0.022 in THA).

**Conclusions:**

These findings indicate that prophylaxis with fondaparinux, not enoxaparin, reduces the risk of DVT but increases bleeding tendency in patients undergoing TKA and THA.

**Trial registration:**

University Hospital Medical Information Network Clinical Trials Registry: UMIN000001366. Registered 11 September 2008.

## Introduction

Postoperative venous thromboembolism (VTE), consisting of deep vein thrombosis (DVT) and pulmonary embolism (PE), is a major life-threating complication in patients undergoing surgery [1]. Patients undergoing total hip and knee arthroplasty (THA and TKA, respectively) are at high risk of VTE [2]. In patients undergoing THA or TKA, thromboprophylaxis with low-molecular-weight heparin (LMWH) or factor Xa inhibitors is recommended for a minimum of 10 to 14 days up to 35 days in current evidence-based guidelines [3,4]. However, it is not clear whether these regimens affect important patient outcomes in “real-world” settings [5]. Despite the need for effective and safe thromboprophylactic drugs in patients undergoing joint replacement therapy, few real-world data assessed the benefits of anticoagulants in this population. The translation of evidence-based guidelines into everyday clinical practice is not immediate [6]. Adoption of these recommendations in patients undergoing elective orthopedic surgery depends on combinations of patient-associated factors, including age, gender, and comorbidities, and physician-associated factors, including clinical practice and institutional compliance, as well as the widespread dissemination of guidelines and educational initiatives to reinforce published guidelines [7,8]. Clinical trials in highly selected patients must be supplemented by efficacy and safety data in “real-world” settings. These evidence gaps have led to differences among recent recommendations; although these guidelines recommend thromboprophylaxis after joint replacement, their choices of optimal prophylactic agents differ [9,10].

In Japan, fondaparinux (June 2007) and enoxaparin (April 2008) had been approved for thromboprophylaxis in patients undergoing THA, TKA, or hip fracture surgery [11,12]. Although these agents are widely used throughout Japan, their effectiveness remains unclear. The Japanese study of Prevention and Actual situation of Venous Thromboembolism after Total Arthroplasty (J-PSVT) is a nationwide multicenter cohort study designed to gain insights into the effectiveness (that is, the prevention of VTE) and safety (that is, the incidence of bleeding and other adverse events) of the “real-world” prophylaxis of VTE after joint replacement surgery in 34 Japanese National Hospital Organization (NHO) hospitals. The main objective of this study was to determine practice patterns of VTE prophylaxis and their outcomes, including symptomatic and non-symptomatic VTEs, bleeding events, and all-cause mortality, in Japanese patients undergoing total joint replacement.

## Materials and methods

### Study design

The J-PSVT is a hospital-based, prospective cohort study designed to document the effectiveness and safety of current standard thromboprophylactic agents, including unfractionated heparin, LMWH, fondaparinux, and anti-platelet agents, approved for use in Japan. Data were collected prospectively on all patients undergoing primary TKA and THA in 34 NHO hospitals since 2007 to 2010. The primary aim of the J-PSVT was to determine the rates of VTEs in patients undergoing TKA and THA, and all patients were evaluated for the presence of all (symptomatic/non-symptomatic) DVT on postoperative day 10 (POD10). Data on patient demographics, primary diagnosis, pre-existing comorbid conditions, length of operation, type of anesthesia, VTE prophylaxis (including type and duration), and mechanical VTE prophylaxis were gathered by using standard case report forms. The trial was registered in the Japan UMIN Clinical Trial Registry (UMIN000001366). The study protocol was approved by the ethics committees of the National Hospital Organization central institutional review board (#0623004). Written informed consent was obtained from each individual for their clinical records to be used in this study.

### Patient enrollment

Patients who were at least 20 years old were eligible if they were scheduled for knee or hip replacement surgery for primary joint diseases, such as osteoarthritis and rheumatoid arthritis. Patients were excluded if they had (1) a predefined risk factor for bleeding (for example, gastrointestinal ulcer and hemorrhagic stroke), (2) a coagulation disorder, (3) heart failure (New York Heart Association class III or IV), (4) renal impairment (creatinine clearance rate of less than 30 mL/min), or (5) liver dysfunction: aspartate transaminase (AST) or alanine transaminase (ALT) of at least five times the upper limit of normal or total bilirubin of at least two times the upper limit of normal. Patients were also excluded if they had undergone joint replacement within 3 months prior to hospital admission, were scheduled to undergo bilateral joint replacement, were unable to walk, or had any severe illness.

### Outcome measures

There were two primary effectiveness outcomes: the composite incidence of asymptomatic/symptomatic DVT up to POD10 and the incidence of fatal/non-fatal PE up to POD28. All enrolled patients were assessed for DVT on POD10, or earlier if thrombosis was clinically suspected, by standard Doppler ultrasonography. DVT diagnosis required confirmation of the presence of a venous thrombus by compression ultrasonography (CUS) [13] performed by using a standardized method [14-16]. All sonographers were adequately trained. Just before the start of study, the participating sonographers received detailed instructions for standardized procedures in a specially organized conference and required a certification process. All patients underwent CUS performed by registered sonographers who were unaware of both risk factors and thromboprophylaxis. The minimal requirement for B-mode ultrasonography was a high-resolution real-time scanner equipped with a 5-MHz electronically focused linear-array transducer, although an ultrasonography device with better specifications (including Doppler and color-Doppler options) was allowed. If Doppler or color-Doppler techniques were used, it was critical that the B-mode pictures allowed the vein walls to be clearly identified. The single criterion indicating the presence of a venous thrombosis was failure to fully compress the venous lumen despite firm compression with the transducer probe. DVT was classified as being in either a proximal (that is, the popliteal vein or any vein proximal to it) or a distal (that is, any vein distal to the popliteal vein) vein. A pulmonary embolism was defined as definite if computed tomography/angiography of the chest or ventilation-perfusion scintigraphy showed a characteristic intraluminal filling defect. The primary safety outcomes were the incidences of major bleeding and death from all causes up to POD28. Major bleeding was defined as hemorrhage occurring at a critical site (for example, intracranial hemorrhage), resulting in the need for a major therapeutic intervention (for example, surgery), causing hemodynamic compromise, requiring at least one unit of red-cell concentrates, or resulting in death. Minor bleeding was defined as bleeding that did not fulfill the criteria for major bleeding.

#### Data collection

Data from all participating doctors were entered into the J-PSVT database at the data center of the NHO headquarters Center for Support and Education of Clinical Research via the HospNet internet system. Previous comorbid conditions of each patient were reviewed by each of the principal physicians. These conditions included cancer, stroke, chronic kidney disease, diabetes, hyperlipidemia, arrhythmia, and cardiovascular diseases as well as previous thrombosis. Medical information collected during follow-up included the development of cardiovascular events, including DVT, myocardial infarction, pulmonary embolism, heart failure, arrhythmia, and cerebrovascular accident as well as gastrointestinal bleeding and death.

### Statistical analysis

Discrete variables were compared by using chi-squared tests and continuous variables by using Kruskal-Wallis rank tests. Multiple logistic regression analysis was used to estimate the risk factors independently associated with the primary composite outcome: asymptomatic/symptomatic DVT up to POD10 and PE up to POD28. Age, sex, history of venous thrombosis, epidermal growth factor receptor (less than 60 mL/min per 1.73 m^2^ versus at least 60 mL/min per 1.73 m^2^), body mass index (at least 30 kg/m^2^ versus less than 30 kg/m^2^), anesthesia (general versus others), operation time (at least 120 minutes versus less than 120 minutes), foot pump use (yes versus no), elastic stocking use (yes versus no), and different anti-coagulating agents (none versus unfractionated heparin (UFH), enoxaparin, fondaparinux, and other agents) were treated as independent variables. Multivariate logistic regression was used to calculate odds ratios and 95% confidence intervals (CIs) after controlling simultaneously for potential confounders. Individuals with missing data were excluded from the model.

Propensity score matching [17] was performed to minimize any effects of confounding caused by non-randomized assignment to VTE prophylaxis. Propensity score matching is used to reduce the impact of selection bias and allows relevant variables to be balanced in patients treated with fondaparinux or enoxaparin for VTE prophylaxis. Logistic regression was used to estimate a propensity score, representing the risk for VTE. Propensity scores were matched 1:1 in patients taking fondaparinux and enoxaparin to compare patients at equivalent risks for VTE. After matching, we compared the incidence of the primary outcome and major bleeding events after surgery. The ratio of risk in the fondaparinux compared with the enoxaparin was calculated by using chi-squared tests. All reported *P* values were two-tailed. All data processing and analysis were performed by using the Statistical Analysis System (SAS) and SPSS version 18 software (SPSS, Chicago, IL, USA).

## Results

### Study populations and treatments

**Figure 1:**
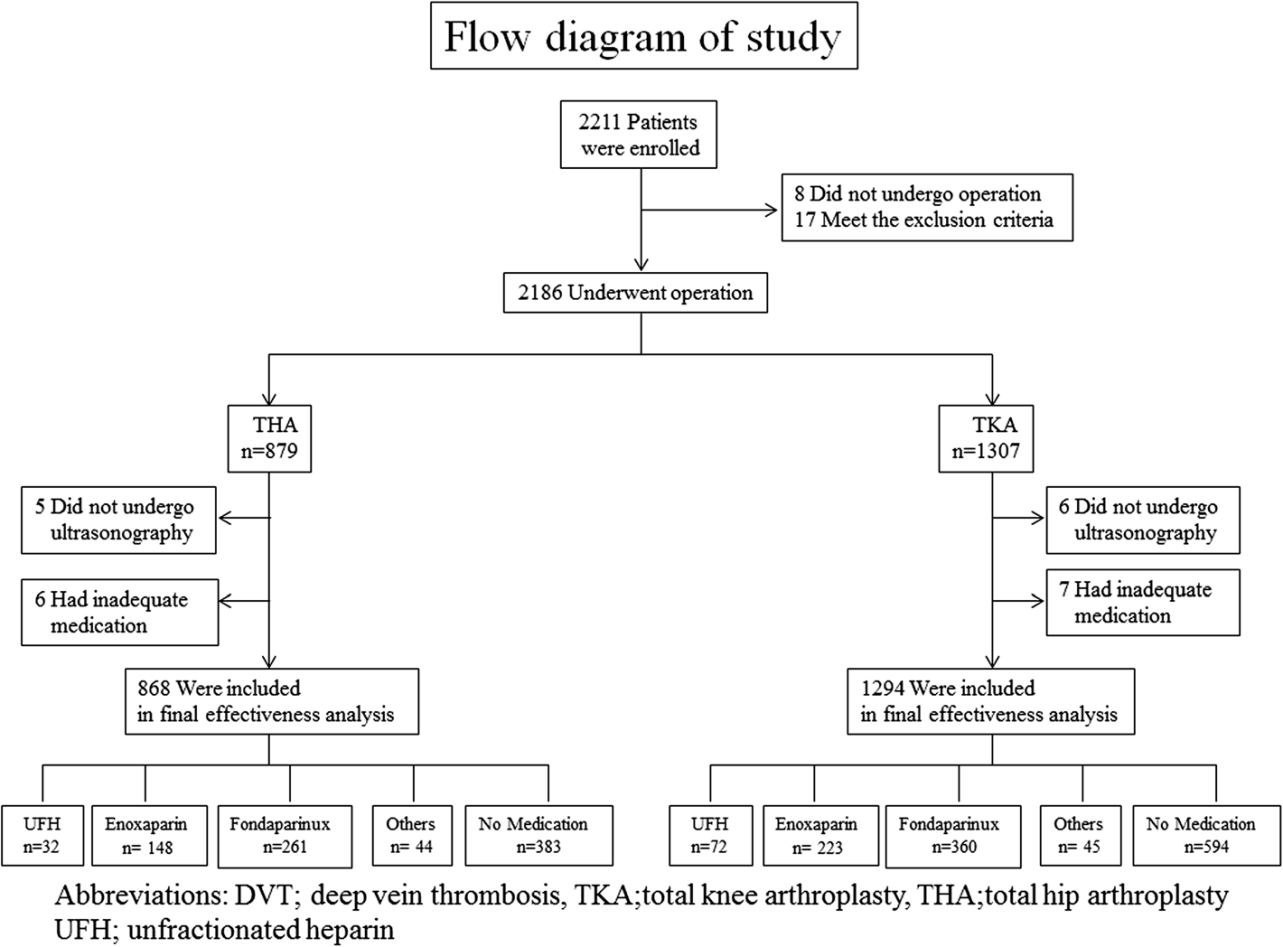
Flow diagram of patient enrollment.

Between 1 July 2007 and 31 May 2010, 2,211 patients undergoing THA or TKA at 34 NHO hospitals were screened for eligibility. Of these, 25 patients were excluded: 17 with exclusion criteria and 8 who cancelled surgery. Of the 2,186 enrolled patients, 1,307 underwent TKA and 879 patients underwent THA. Primary effectiveness outcome was not assessed in 13 patients who underwent TKA and in 11 who underwent THA owing to the lack of ultrasonography or inadequate medical treatments. Thus, the overall study population consisted of 1,294 patients who underwent TKA and 868 patients who underwent THA (Figure 1).

The demographic and clinical characteristics of these patients are shown in Table 1. VTE prophylaxis showed apparent variations. Most physicians in Japan prescribe more than one modality (mechanical and pharmacological) in combination, with foot pumps and elastic stockings used in combination with pharmacological prophylaxis in many of these patients. Pharmacological thromboprophylaxis in patients undergoing TKA consisted of fondaparinux in 360 patients (27.8%), enoxaparin in 223 (17.2%), UFH in 72 (5.6%), and anti-platelet agents (others: aspirin 41, ticlopidine 2, and cilostazol 2) in 45 (3.5%), whereas 594 patients (45.9%) received no medication. Among these 594 patients without medication, 591 patients (99.5%) received mechanical thromboprophylaxis (elastic stocking 36.5%, foot pump 15.0%, and elastic stocking plus foot pump 48.0%). These devices were initiated post-operatively, and the same type of elastic stockings were used in both patients receiving TKA and THA. Among patients undergoing THA, 261 (30.1%) were treated with fondaparinux, 148 (17.1%) with enoxaparin, 32 with UFH (3.7%) anti-platelet agents (others: aspirin 42 and cilostazol 2) in 44 (5.1%), and 383 (44.1%) with no medication. Among 383 patients without medication, 381 patients (99.5%) received mechanical thromboprophylaxis (elastic stocking 40.2%, foot pump 7.8%, and elastic stocking plus foot pump 51.4%). Patients weighing less than 50 kg and at least 50 kg received once-daily subcutaneous injection of 1.5 and 2.5 mg fondaparinux, respectively, or twice-daily subcutaneous injections of 2,000 international units (IU) of enoxaparin sodium (Clexane, Sanofi, Paris, France). Fondaparinux was initiated after 24 hours from wound closure, and enoxaparin after 24 to 36 hours. UFH was initiated after 6 to 12 hours from wound closure. The mean doses (lengths) of thromboprophylaxis with fondaparinux, enoxaparin, and UFH were 1.7 ± 0.5 mg (10.2 ± 7.2 days), 2,854 ± 992 IU (8.1 ± 4.4 days), and 13,911 ± 101 units (5.8 ± 5.8 days), respectively, in patients undergoing TKA and 1.8 ± 0.5 mg (9.5 ± 4.0 days), 3,635 ± 775 IU (8.4 ± 3.9 days), and 10,133 ± 2,162 units (5.1 ± 3.0 days), respectively, in patients undergoing THA. Treatment with anti-platelet agents before the day of surgery was considered a continuation of thromboprophylaxis, and these patients were excluded (inadequate medical treatment).

**Table 1:** Baseline and other characteristics of the study patients

	**TKA (n = 1,294)**	**THA (n = 868)**
Gender, male/female	221/1,073	128/740
Age, years		
Mean ± SD	73.9 ± 8.0	66.7 ± 10.5
Range	34-93	23-94
Weight, kg		
Mean ± SD	57.8 ± 10.6	54.8 ± 11.4
Body mass index, kg/m^2^		
Mean ± SD	25.4 ± 3.9	23.6 ± 4.2
Range	14.5-44.0	14.2-56.4
History of venous thrombolism, n (%)	16 (1.2%)	5 (0.6%)
Comorbidities, n (%)		
Hypertension	706 (54.6%)	307 (35.4%)
Ischemic heart disease	75 (5.8%)	40 (4.6%)
Diabetes	194 (15.0%)	69 (7.9%)
Cerebrovascular disease	63 (4.9%)	24 (2.8%)
Operation time, minutes		
Mean ± SD	127 ± 37.2	123.3 ± 43.0
Range	42-400	35-332
Type of anesthesia, n (%)		
General	366 (28.3%)	280 (32.3%)
Epidural	56 (4.3%)	31 (3.6%)
Spinal	337 (26.0%)	88 (10.1%)
General + epidural	535 (41.3%)	469 (54.0%)
Primary diseases, n (%)		
Rheumatoid arthritis	204 (15.8%)	52 (6.0%)
Osteoarthritis	1,084 (83.8%)	751 (86.5%)
Others	6 (0.5%)	65 (7.5%)
Use of elastic stocking, n (%)	1,095 (84.6%)	792 (91.2%)
Use of foot pump, n (%)	939 (72.6%)	669 (77.1%)
Use of tourniquet, n (%)	1,244 (96.1%)	-
Use of cement, n (%)	1,208 (93.4%)	183 (21.1%)
Prophylaxis		
Unfractionated heparin, n (%)	72 (5.6%)	32 (3.7%)
Enoxaparin, n (%)	223 (17.2%)	148 (17.1%)
Fondaparinux, n (%)	360 (27.8%)	261 (30.1%)
Others, n (%)	45 (3.5%)	44 (5.1%)
No medication, n (%)	594 (45.9%)	383 (44.1%)
Laboratory data		
Serum albumin, g/dL		
Mean ± SD	4.1 ± 0.4	4.1 ± 0.4
Hemoglobin, g/dL		
Mean ± SD	12.4 ± 1.6	12.1 ± 1.5
Platelet count, 10^4^/μL		
Mean ± SD	24.5 ± 14.3	26.4 ± 11.1
e-GFR, mL/min per 1.73 m^2^		
Mean ± SD	73.4 ± 21.4	80.8 ± 20.1

e-GFR, estimated glomerular filtrating ratio; SD, standard deviation; THA, total hip arthroplasty; TKA, total knee arthroplasty.

### Incidence of VTE and major bleeding

No mortality was confirmed in this study. Three events of cardiovascular disease (cerebral infarction) were observed during the follow-up periods (up to POD28).

The incidence of the composite of symptomatic and non-symptomatic DVT differed in patients undergoing TKA and THA. The overall rate of DVT through POD10 in patients undergoing TKA (Table 2) was 24.3% (314 patients), and symptomatic DVT occurred in 12 patients (0.9%). PEs were confirmed in one patient treated with fondaparinux (day 7) and in one patient with no medication (day 13). In patients undergoing THA (Table 3), the overall rate of DVT up to POD10 was 12.6% (109 patients), and symptomatic DVT occurred in two patients (0.2%) and none experienced PE. Symptomatic DVT over the POD10 and within POD28 was found in two patients undergoing TKA and one patient undergoing THA.

**Table 2:** Incidences of primary effectiveness outcomes in patients receiving TKA

	**Fondaparinux**	**Enoxaparin**	**UFH**	**Others**	**Medication (-)**	**Total**
	**n = 360 (%)**	**n = 223 (%)**	**n = 72 (%)**	**n = 45 (%)**	**n = 594 (%)**	**n = 1,294 (%)**
All venous thromboembolism	61 (16.9)	59 (26.5)	24 (33.3)	12 (26.7)	158 (26.6)	314 (24.3)
Any DVT (up to POD10 )	60 (16.7)	59 (26.5)	24 (33.3)	12 (26.7)	158 (26.6)	314 (24.3)
Symptomatic DVT	2 (0.6)	3 (1.3)	0	1 (2.2)	6 (1.0)	12 (0.9)
Distal	1 (0.3)	2 (0.9)	0	1 (2.2)	4 (0.7)	8 (0.6)
Proximal	1 (0.3)	1 (0.4)	0	0	2 (0.3)	4 (0.3)
Non-symptomatic DVT	59 (16.4)	56 (25.1)	24 (33.3)	11 (24.4)	152 (25.6)	302 (23.3)
Distal	51 (14.2)	52 (23.3)	21 (29.2)	11 (24.4)	133 (22.4)	268 (20.7)
Proximal	8 (2.2)	4 (1.8)	3 (4.2)	0	19 (3.2)	34 (2.6)
PE (up to POD28)	1 (0.3)	0	0	0	1 (0.2)	2 (0.2)

CI, confidence interval; DVT, deep vein thrombosis; PE, pulmonary embolism; POD, post-operative day; TKA, total knee arthroplasty; UFH, unfractionated heparin.

**Table 3:** Incidences of primary effectiveness outcomes in patients receiving THA

	**Fondaparinux**	**Enoxaparin**	**UFH**	**Others**	**Medication (-)**	**Total**
	**n = 261 (%)**	**n = 148 (%)**	**n = 32 (%)**	**n = 44 (%)**	**n = 383 (%)**	**n = 868 (%)**
All venous thromboembolism	17 (6.5)	17 (11.5)	5 (15.6)	8 (18.2)	62 (16.2)	109 (12.6)
Any DVT (up to POD10 )	17 (6.5)	17 (11.5)	5 (15.6)	8 (18.2)	62 (16.2)	109 (12.6)
Symptomatic DVT	0	1 (0.7)	0	0	1 (0.3)	2 (0.2)
Distal	0	1 (0.7)	0	0	1 (0.3)	2 (0.2)
Proximal	0	0	0	0	0	0
Non-symptomatic DVT	17 (6.5)	16 (10.8)	5 (15.6)	8 (18.2)	61 (15.9)	107 (12.3)
Distal	15 (5.7)	13 (8.8)	3 (9.4)	8 (18.2)	51 (13.3)	90 (10.4)
Proximal	2 (0.8)	3 (2.0)	2 (6.3)	0	10 (2.6)	17 (2.0)
PE (up to POD28)	0	0	0	0	0	0

CI, confidence interval; DVT, deep vein thrombosis; PE, pulmonary embolism; POD, post-operative day; THA, total hip arthroplasty; UFH, unfractionated heparin.

DVT rates varied among patients receiving different patterns of pharmacologic thromboprophylaxis. The overall rates of DVT up to POD10 in patients undergoing TKA were 16.7% with fondaparinux, 26.5% with enoxaparin, 33.3% with UFH, 26.7% with other medications, and 26.6% with no medication (Table 2). The overall rates of DVT up to POD10 in patients undergoing THA were 6.5% with fondaparinux, 11.5% with enoxaparin, 15.6% with UFH, 18.2% with other medications, and 16.2% with no medication (Table 3). Safety analysis showed that the incidences of major bleeding up to POD28 in patients undergoing TKA and THA were 1.2% (n = 15) and 2.3% (n = 20), respectively. Major or minor bleeding occurred at early periods postoperatively in both patients receiving TKA (major 2.7 ± 3.5 days, minor 5.0 ± 2.7 days) and THA (major 2.6 ± 2.9 days, minor 5.6 ± 3.0 days). Major bleeding was also confirmed in patients without pharmacologic thromboprophylaxis (TKA 5 patients 33.3%, THA 7 patients 35.0%). No fatal bleeding was observed (Table 4).

**Table 4:** Incidences of bleeding in patients receiving joint replacement

	**TKA**	**THA**
	**n = 1,294 (%)**	**n = 868 (%)**
All bleeding events, n (%)	46/1,294 (3.6)	31/868 (3.6)
Major bleeding	15/1,294 (1.2)	20/868 (2.3)
Bleeding in critical organ	1/1,294 (0.1)	0/868 (0)
Bleeding leading to reoperation	0/1,294 (0)	0/868 (0)
Bleeding requiring of ≧1 unit of transfusion	14/1,294 (1.1)	20/868 (2.3)
Bleeding contributing to death	0/1,294 (0)	0/868 (0)
Minor bleeding	31/1,294 (2.4)	11/868 (1.3)

THA, total hip arthroplasty; TKA, total knee arthroplasty.

### Risk factors for postoperative DVT

Univariate analysis isolated several variables which are significantly different between patients with or without the primary effectiveness outcomes (Tables 5 and 6). Multivariate analyses were performed to identify independent predictors of the primary effectiveness outcomes (Tables 5 and 6). Increased VTE rates were significantly associated with female gender and the use of foot pumps in patients undergoing TKA (Table 7) and with female gender and older age in patients undergoing THA (Table 8). The use of general anesthesia was significantly associated with reduced risks of VTE in both groups. Among pharmacological prophylaxis, only prophylaxis with fondaparinux was significantly associated with reduced risks of VTE in both groups.

**Table 5:** Baseline characteristics of patients with or without DVT in patients receiving TKA

	**DVT (+)**	**DVT (-)**	***P *** **value**
	**n = 314**	**n = 980**	
Age, ≧75 years	185 (58.9%)	512 (52.2%)	0.039
Female gender	274 (87.3%)	799 (81.5%)	0.019
History of venous thrombosis	6 (1.9%)	10 (1.0%)	0.169
e-GFR, <60 mL/min per 1.73 m^2^	86 (27.5%)	245 (25.1%)	0.392
Body mass index, ≧30 kg/m^2^	31 (9.9%)	118 (12.1%)	0.287
General anesthesia	199 (63.4%)	702 (71.6%)	0.006
Use of tourniquet	308 (98.1%)	936 (95.5%)	0.039
Operation time, ≧120 minutes	167 (53.2%)	562 (57.3%)	0.196
Use of foot pump	241 (76.8%)	698 (71.2%)	0.056
Use of elastic stocking	267 (85.0%)	828 (84.5%)	0.817
Medications			
Unfractionated heparin	24 (7.6%)	48 (4.9%)	0.065
Enoxaparin	59 (18.8%)	164 (16.7%)	0.401
Fondaparinux	61 (19.4%)	299 (30.5%)	<0.0001
Others	12 (3.8%)	33 (3.4%)	0.702

CI, confidence interval; DVT, deep vein thrombosis; e-GFR, estimated glomerular filtrating ratio; PE, pulmonary embolism; TKA, total knee arthroplasty.

**Table 6:** Baseline characteristics of patients with or without DVT in patients receiving THA

	**DVT(+)**	**DVT(-)**	***P *** **value**
	**n = 109**	**n = 759**	
Age, ≧75 years	43 (39.4%)	189 (24.9%)	0.001
Female gender	102 (93.6%)	638 (84.1%)	0.009
e-GFR, <60 mL/min per 1.73 m^2^	17 (15.6%)	102 (13.5%)	0.547
Body mass index, ≧30 kg/m^2^	7 (6.4%)	49 (6.5%)	0.981
General anesthesia	83 (76.1%)	666 (87.7%)	0.001
Operation time, ≧120 minutes	42 (38.5%)	353 (46.5%)	0.118
Use of foot pump	83 (76.1%)	586 (77.2%)	0.806
Use of elastic stocking	95 (87.2%)	697 (91.8%)	0.106
Medications			
Unfractionated heparin	5 (4.6%)	27 (3.6%)	0.374
Enoxaparin	17 (15.6%)	131 (17.3%)	0.666
Fondaparinux	17 (15.6%)	244 (32.1%)	<0.0001
Others	8 (7.3%)	36 (4.7%)	0.248

CI, confidence interval; DVT, deep vein thrombosis; e-GFR, estimated glomerular filtrating ratio; PE, pulmonary embolism; THA, total hip arthroplasty.

**Table 7:** **Multivariate logistic regression for the composite effectiveness outcomes (symptomatic/non-symptomatic DVT up to POD10 and PE up to POD28) in patients receiving TKA**

**Predictors**	**Odds ratio**	**95% CI**	***P *** **value**
Age, ≧75 years	1.30	0.99-1.71	0.060
Female gender	1.58	1.08-2.31	0.018
History of venous thrombosis	1.11	0.36-3.46	0.846
e-GFR, <60 mL/min per 1.73 m^2^	1.05	0.77-1.42	0.768
Body mass index, ≧30 kg/m^2^	0.90	0.58-1.38	0.626
General anesthesia	0.60	0.45-0.80	0.001
Use of tourniquet	2.31	0.95-5.59	0.065
Operation time, ≧120 minutes	0.91	0.69-1.21	0.504
Use of foot pump	1.60	1.15-2.21	0.005
Use of elastic stocking	1.28	0.85-1.94	0.234
Medications			
Unfractionated heparin	1.61	0.92-2.79	0.093
Enoxaparin	1.01	0.71-1.45	0.961
Fondaparinux	0.51	0.36-0.72	0.000
Others	1.00	0.49-2.02	0.992

CI, confidence interval; DVT, deep vein thrombosis; e-GFR, estimated glomerular filtrating ratio; PE, pulmonary embolism; TKA, total knee arthroplasty.

**Table 8:** **Multivariate logistic regression for the composite effectiveness outcomes (symptomatic/non-symptomatic DVT up to POD10 and PE up to POD28) in patients receiving THA**

**Predictors**	**Odds ratio**	**95% CI**	***P *** **value**
Age, ≧75 years	1.82	1.15-2.87	0.011
Female gender	2.31	1.03-5.18	0.041
e-GFR, <60 mL/min per 1.73 m^2^	0.81	0.44-1.50	0.502
Body mass index, ≧30 kg/m^2^	0.89	0.36-2.20	0.803
General anesthesia	0.41	0.23-0.71	0.002
Operation time, ≧120 minutes	0.79	0.50-1.23	0.293
Use of foot pump	1.08	0.59-1.96	0.808
Use of elastic stocking	0.51	0.26-1.01	0.052
Medications			
Unfractionated heparin	1.27	0.44-3.61	0.658
Enoxaparin	0.84	0.44-1.57	0.577
Fondaparinux	0.36	0.20-0.66	0.001
Others	1.36	0.55-3.36	0.502

CI, confidence interval; DVT, deep vein thrombosis; e-GFR, estimated glomerular filtrating ratio; PE, pulmonary embolism; THA, total hip arthroplasty.

### Effectiveness and safety of fondaparinux versus enoxaparin in propensity score-matched analysis

To date, no randomized controlled trial has directly compared fondaparinux and enoxaparin for thromboprophylaxis in Japanese patients. We therefore used propensity score matching to adjust for differences in baseline risks to patients treated with these agents. We matched equal numbers of patients receiving fondaparinux and enoxaparin, with no significant between group differences, except for the rates of general anesthesia (TKA), the use of foot pump (TKA), and serum albumin concentrations (TKA and THA), in baseline characteristics (Tables 9 and 10). Tables 11 and 12 show the overall rates of DVT and major bleeding in the propensity score-matched patients undergoing TKA (Table 11) and THA (Table 12). The incidence of any DVT up to POD10 was lower with fondaparinux than with enoxaparin, both in patients undergoing TKA (13.6% versus 26.2%) and THA (5.6% versus 11.1%). However, the rate of major bleeding events was higher in fondaparinux- than in enoxaparin-treated patients in both groups.

**Table 9:** Baseline and other characteristics of the propensity score-matched patients receiving fondaparinux or enoxaparin (TKA)

	**Fondaparinux**	**Enoxaparin**	***P *** **value**
	**n = 204**	**n = 204**	
Gender, male/female	30/174	26/178	0.565
Age, years			
Mean ± SD	73.6 ± 7.6	73.2 ± 7.7	0.734
Range	34-87	45-89	
Body mass index, kg/m^2^			
Mean ± SD	25.4 ± 4.0	25.5 ± 4.0	0.729
History of venous thrombosis, n (%)	3 (1.5%)	2 (1.0%)	0.500
Comorbidities, n (%)			
Hypertension	113 (55.4%)	98 (48.0%)	0.137
Ischemic heart disease	9 (4.4%)	11 (5.4%)	0.647
Diabetes	29 (14.2%)	27 (13.2%)	0.774
Cerebrovascular disease	11 (5.4%)	7 (3.4%)	0.335
Operation time, minutes			
Mean ± SD	131.4 ± 41.7	130.2 ± 46.7	0.786
Primary diseases, n (%)			
Rheumatoid arthritis	41 (20.1%)	27 (13.2%)	0.063
Osteoarthritis	161 (78.9%)	176 (86.3%)	0.050
Others	2 (1.0%)	1 (0.5%)	0.500
General anesthesia	175 (85.8%)	150 (73.5%)	0.002
Use of elastic stocking, n (%)	195 (95.6%)	184 (90.2%)	0.034
Use of foot pump, n (%)	184 (90.2%)	156 (76.5%)	<0.0001
Use of tourniquet, n (%)	187 (91.7%)	193 (94.6%)	0.240
Use of cement, n (%)	198 (97.1%)	198 (97.1%)	1.000
e-GFR, mL/min per 1.73 m^2^			
Mean ± SD	74.7 ± 22.1	74.2 ± 19.4	0.961
Range	24.1-158.7	30.9-147.5	
Serum albumin, g/dL			
Mean ± SD	4.0 ± 0.5	4.1 ± 0.4	0.041
Range	2.3-5.0	2.8-4.9	
Platelet count, 10^4^/μL			
Mean ± SD	24.8 ± 6.9	24.9 ± 7.2	0.913

Kruskal-Wallis rank test were performed on the means of continuous variables. For categorical variables, chi-squared tests were used. e-GFR, estimated glomerular filtrating ratio; SD, standard deviation; TKA, total knee arthroplasty.

**Table 10:** Baseline and other characteristics of the propensity score-matched patients receiving fondaparinux or enoxaparin (THA)

	**Fondaparinux**	**Enoxaparin**	***P *** **value**
	**n = 144**	**n = 144**	
Gender, male/female	20/124	23/121	0.620
Age, years			
Mean ± SD	67.3 ± 10.0	68.0 ± 8.8	0.707
Range	40-87	44-86	
Body mass index, kg/m^2^			
Mean ± SD	23.4 ± 3.8	23.8 ± 3.6	0.386
History of venous thrombosis, n (%)	-	-	
malignant tumor	5 (3.5%)	0	0.030
Comorbidities, n (%)			
Hypertension	59 (41.0%)	54 (37.5%)	0.546
Ischemic heart disease	7 (4.9%)	6 (4.2%)	0.777
Diabetes	11 (7.6%)	10 (6.9%)	0.821
Cerebrovascular disease	4 (2.8%)	3 (2.1%)	0.702
Operation time, minutes			
Mean ± SD	124.6 ± 38.7	125.6 ± 47.3	0.931
Primary diseases, n (%)			
Rheumatoid arthritis	9 (6.3%)	5 (3.5%)	0.273
Osteoarthritis	129 (89.6%)	129 (89.6%)	1.000
Others	6 (4.2%)	10 (6.9%)	0.303
General anesthesia	141 (97.9%)	141 (97.9%)	1.000
Use of elastic stocking, n (%)	131 (91.0%)	133 (92.4%)	0.670
Use of foot pump, n (%)	138 (95.8%)	137 (95.1%)	0.777
Use of cement, n (%)	23 (16.0%)	24 (16.7%)	0.873
e-GFR, mL/min per 1.73 m^2^			
Mean ± SD	81.9 ± 20.0	78.6 ± 16.8	0.220
Range	29.0-139.0	37.7-128.9	
Serum albumin, g/dL			
Mean ± SD	4.0 ± 0.5	4.1 ± 0.4	0.001
Range	2.4-5.0	2.6-5.1	
Platelet count, 10^4^/μL			
Mean ± SD	27.7 ± 7.6	26.0 ± 7.7	0.048

Kruskal-Wallis rank test were performed on the means of continuous variables. For categorical variables, chi-squared tests were used. e-GFR, estimated glomerular filtrating ratio; SD, standard deviation; THA, total hip arthroplasty.

**Table 11:** Incidences of any DVT (up to POD10) and major bleeding (up to POD28) in propensity-matched fondaparinux- or enoxaparin-treated groups (TKA)

**Events**	**Fondaparinux**	**Enoxaparin**	**Risk ratio (95% CI)**	***P *** **value**
	**n = 204**	**n = 204**		
DVT	28/204 (13.6%)	54/204 (26.2%)	0.70 (0.58-0.85)	0.002
Major bleeding	7/204 (3.4%)	1/204 (0.5%)	4.18 (0.67-26.20)	0.062

CI, confidence interval; DVT, deep vein thrombosis; POD, postoperative day; TKA, total knee arthroplasty.

**Table 12:** Incidences of any DVT (up to POD10) and major bleeding (up to POD28) in propensity-matched fondaparinux- or enoxaparin-treated groups (THA)

**Events**	**Fondaparinux**	**Enoxaparin**	**Risk ratio (95% CI)**	***P *** **value**
	**n = 144**	**n = 144**		
DVT	8/144 (5.6%)	16/144 (11.1%)	0.73 (0.53-0.99)	0.134
Major bleeding	7/144 (4.9%)	0/144 (0%)	-	0.022

CI, confidence interval; DVT, deep vein thrombosis; POD, postoperative day; THA, total hip arthroplasty.

## Discussion

Patients undergoing joint replacement surgery require effective thromboprophylaxis with both pharmaceutical agents and mechanical methods [3,4]. Despite these measures, however, approximately 10% to 15% of patients experience subclinical DVT after surgery, and 0.5% to 2.0% have symptomatic VTE during the first 3 months after surgery [18,19]. Thromboprophylactic agents approved in Japan include enoxaparin, fondaparinux [11,12], and the oral factor Xa inhibitor edoxaban, which was approved after the start of this study [20], all of which are clinically used in real-world settings [21]. Translating the efficacy and safety of an agent from a clinical trial to real-world practice is often challenging because participants in trials are usually younger and have less medically complex illnesses. Because the risks of both thrombosis and hemorrhage increase substantially with age and with the burden of chronic diseases, the effectiveness of newly approved agents in real-world settings should be carefully monitored, particularly among older patients [22].

We have assessed the rates of symptomatic/non-symptomatic VTEs and bleeding in a real-world setting of patients undergoing TKA and THA while receiving various thromboprophylactic regimens. We found that the overall rates of asymptomatic and symptomatic DVT up to POD10 and PE up to POD28 were 23.3%, 0.9%, and 0.2%, respectively, in patients undergoing TKA and 12.3%, 0.2%, and 0%, respectively, in patients undergoing THA. Pharmacological prophylaxis was administered to 54% of patients undergoing TKA and 56% undergoing THA. However, multivariate analysis showed that treatment with fondaparinux, but not enoxaparin, reduced VTE (up to POD10). In addition, multivariate analysis showed that risk factors for postoperative VTE included older age (more than 75 years) and female gender, both of which were previously shown to be risk factors for VTEs [23]. We also found that intermittent plantar compression (foot pumps) was an independent risk factor for VTE in patients undergoing TKA, a finding not previously shown. When we directly compared DVT and bleeding in the propensity score-matched fondaparinux- and enxoparin-treated groups, we found that, compared with enoxaparin, fondaparinux reduced the risk of DVT but increased the risk of major bleeding.

Adherence to treatment guidelines can vary by country and clinical practice. A recent publication by the Global Orthopedic Registry (GLORY) reported that only 62% and 69% of patients undergoing THR and TKR, respectively, complied with American College of Chest Physicians guidelines [24]. In comparison, 56% and 54% of patients in our J-PSVT registry who underwent THA and TKA, respectively, received pharmacological thromboprophylaxis. A recent meta-analysis of symptomatic VTE rates prior to hospital discharge, in patients receiving thromboprophylaxis with fondaparinux, LMWH, or oral Xa inhibitors for TKA and THA, found that the VTE rates in patients undergoing TKA and THA were 1.09% and 0.53%, respectively [25]. We found that the rates of overall and symptomatic DVT were lower in patients undergoing TKA (24.3% and 0.9%, respectively) and in patients undergoing THA (12.6% and 0.2%, respectively). Interpretation of DVT rate in patients who underwent orthopedic surgery may be affected by differences in time frames, methods of diagnosing DVT, and thromboprophylaxis protocol. Additionally, risk factors for VTE in Asian patients may not be different from those of Western patients through the genetic backgrounds. Deficiencies of the natural anti-coagulants (protein C, protein S, and antithrombin) are the predominant thrombophilias in Asia, whereas factor V Leiden and prothrombin G20210A gene mutation are rarely reported [26].

We found that intermittent plantar compression (foot pumps) was an independent risk factor for VTE in patients undergoing TKA, a result not previously reported. Mechanical prophylaxis, both pneumatic compression and intermittent plantar compression (foot pump), has been studied in patients undergoing TKA [27]. Despite showing that mechanical prophylaxis significantly reduced thrombus formation, these studies were low-powered [28,29], indicating the need for large multicenter randomized trials to determine the efficacy of these devices. No consensus has yet been reached on the anesthetic technique optimal for thromboprophylaxis in patients undergoing joint replacement. In contrast to previous results [30], we found that general anesthesia was associated with a reduced risk for VTE. Studies comparing the incidence of DVT in patients undergoing general and epidural anesthesia have yielded contradictory results [31,32]. Moreover, since about half of our patients received both general and local anesthesia, further studies are needed.

Although clinical trials have compared the efficacy and safety of fondaparinux and enoxaparin for preventing VTE in patients undergoing major orthopedic surgery of the lower limbs [33-36], these two agents were never compared directly in Japanese patients. When we compared their effectiveness in a propensity score-matched population, we found that fondaparinux significantly reduced the incidence of ultrasound-proven DVT compared with enoxaparin. In contrast, major bleeding, leading to a requirement for transfusion of at least one unit of blood or occurring in a critical organ, occurred more frequently in the fondaparinux- than in the enoxaparin-treated group. A meta-analysis of four randomized double-blind trials comparing fondaparinux with enoxaparin found that 2.5 mg/day fondaparinux was superior to approved enoxaparin regimens in preventing VTE [37]. Furthermore, the overall incidence of clinical-relevant bleeding did not differ between the two groups, and the benefit of fondaparinux was consistent across all studies. In one trial, however, the rates of major bleeding were significantly higher with fondaparinux than with enoxaparin [36]. The lack of consistency in defining “bleeding” in these studies, including ours, creates difficulties in interpreting the true benefit-harm balance. Pharmacological prophylaxis in patients undergoing major orthopedic surgery is of concern because of the increased risk of bleeding. Several previous studies have found an interaction between dose of fondaparinux and risk of major bleeding [38,39]. Overall, clinicians must make trade-offs between the benefits of reducing thrombosis and adverse effects, including bleeding, when using fondaparinux in Japanese patients.

Some methodological aspects and possible limitations of this study require comment. First, because of the non-interventional, open-label study design and limitations inherent to observational studies, the estimated risks were not unbiased. Owing to a lack of randomization, observational studies are confounded by indication. Although our study cohort was large and the study population was adjusted for a large number of confounding covariates, we could not adjust for all confounders. Thus, this observational study was not the equivalent of a randomized control trial. Second, indications for thromboprophylaxis varied widely among physicians and within hospitals, introducing an inherent selection bias. The incorporation of DVT into the composite primary efficacy end point of this study may be questionable [9]. However, DVT, both symptomatic and non-symptomatic, has been linked with symptomatic or fatal PE [40]. Venography is generally accepted as the gold standard in detecting DVT. A recent systematic review suggested that ultrasound is accurate for the postoperative diagnosis of DVT in asymptomatic orthopedic patients [13]. Additionally, the use of blinded investigators and independent adjudication may reduce some of the imprecision stemming from subjectivity and variability among observers [41]. Although the risk of VTE was shown to be extended by periods beyond the usual periods of hospitalization [42], the duration of pharmacological prophylaxis was relatively limited in our study. The association between mortality and major bleeding is strong in the first 30 days; however, it remains significant up to 3 years [43]. Our study did not determine whether in-hospital bleeding affects the long-term outcomes. Most asymptomatic DVTs detected by using the CUS method were distal, for which the diagnostic performance of CUS is poorer than for proximal DVT [44]. However, CUS yielded much better diagnostic performance in patients with asymptomatic DVT when performed by staff with substantial experience in ultrasonography and when a standardized examination procedure was used [45], as in our study. Even if the relative inaccuracy of CUS for detecting distal DVTs was real in our study, it would not explain the decreased incidences of DVT in patients receiving a certain thromboprophylaxis agent, because the same diagnostic procedure was used in all patients, regardless of thromboprophylactic agent. Propensity scores are estimated by using a large number of measured pretreatment covariates in a multivariate logistic regression model to predict exposure. Thus, propensity score-matched analysis of patients receiving fondaparinux and enoxaparin mimics a randomized trial. However, unmeasured characteristics and confounders are not completely balanced.

Our study represents the most comprehensive, hospital-based cohort study to date, with the outcomes in all enrolled patients completely followed. The participants in this study, in contrast to those in many clinical trials, were similar demographically to the general population undergoing joint replacement, suggesting that our findings are applicable to the general population. The J-PSVT has been able to recruit a large, diverse population of patients undergoing THA or TKA and therefore was able to identify factors affecting outcomes that may not be apparent in clinical trials.

## Conclusions

This large, prospective, multicenter analysis assessed VTE risks and bleeding in patients undergoing joint replacement surgery under conditions reflecting routine “real-world” clinical practice in Japan. Our results suggest that fondaparinux prophylaxis can reduce DVT but that it is accompanied by a high risk of bleeding. These gaps between recommendations and real-world outcomes should be addressed by additional prospective studies or the registry.

## Abbreviations

CUS: compression ultrasonography; DVT: deep vein thrombosis; J-PSVT: Japanese study of Prevention and Actual situation of Venous Thromboembolism after Total Arthroplasty; LMWH: low-molecular-weight heparin; NHO: National Hospital Organization; PE: pulmonary embolism; POD: post-operative day; THA: total hip arthroplasty; TKA: total knee arthroplasty; UFH: unfractionated heparin; VTE: venous thromboembolism.

## Competing interests

SMi received research support and speaker honoraria from Daiichi Sankyo Co., Ltd (Tokyo, Japan), Mitsubishi Tanabe Pharma Corporation (Osaka, Japan), and CSL Behring K.K. (Tokyo, Japan). The other authors declare that they have no competing interests.

## Authors’ contributions

KM, SB, and SMo participated in the design of the study, helped to analyze the data, and helped to write the manuscript. MN and SMi participated in the design of the study. MK helped to analyze the data. MS, HKak, YuN, ToM, IF, YS, TTa, MY, HKan, IA, TaM, KI, SK, KS, HM, TS, YaN, and TTo helped to collect the clinical data. All authors read and approved the final manuscript.
